# Dual-Mode On-to-Off Modulation of Plasmon-Induced Transparency and Coupling Effect in Patterned Graphene-Based Terahertz Metasurface

**DOI:** 10.1186/s11671-019-3237-y

**Published:** 2020-01-02

**Authors:** Zhimin Liu, Enduo Gao, Zhenbin Zhang, Hongjian Li, Hui Xu, Xiao Zhang, Xin Luo, Fengqi Zhou

**Affiliations:** 1 School of Science, East China Jiaotong University, Nanchang, 330013 China; 2 Department of Materials Science and Engineering, The Ohio State University, 2041 College Road, Columbus, OH 43210 USA; 3 School of Physics and Electronics, Central South University, Changsha, 410083 China

**Keywords:** Plasmon-induced transparency, Graphene, Dual-mode on-to-off modulation, Metasurface

## Abstract

The plasmon-induced transparency (PIT), which is destructive interference between the superradiation mode and the subradiation mode, is studied in patterned graphene-based terahertz metasurface composed of graphene ribbons and graphene strips. As the results of finite-difference time-domain (FDTD) simulation and coupled-mode theory (CMT) fitting, the PIT can be dynamically modulated by the dual-mode. The left (right) transmission dip is mainly tailored by the gate voltage applied to graphene ribbons (stripes), respectively, meaning a dual-mode on-to-off modulator is realized. Surprisingly, an absorbance of 50% and slow-light property of 0.7 ps are also achieved, demonstrating the proposed PIT metasurface has important applications in absorption and slow-light. In addition, coupling effects between the graphene ribbons and the graphene strips in PIT metasurface with different structural parameters also are studied in detail. Thus, the proposed structure provides a new basis for the dual-mode on-to-off multi-function modulators.

## Introduction

At present, surface plasmon polaritons (SPPs), as a carrier for transmitting information and energy, have become a research hotspot in the sub-wavelength optics. Generally, they are produced by the interaction between the photons in the incident light field and the electrons on the metal or insulator surface [1, 2]. The SPPs facilitate the development and manufacture of highly integrated optics and photonic circuits owing to their unique optical properties. Firstly, they are non-radiative modes with great near-field enhancement effects. Secondly, the SPPs can break through the traditional optical diffraction limitation and localize the light in the sub-wavelength range [3]. Thirdly, their properties depend on the physical parameters of the surrounding material. Therefore, SPPs-based metal-dielectric-metal (MDM) waveguides have been widely studied by scholars owing to their low-bending loss, strong local capability, and low manufacturing difficulty. At the same time, many types of MDM plasmonic waveguides have been proposed, such as splitters [4, 5], demultiplexers [6, 7], filters [8–10], and sensors [11, 12]. However, it is particularly inconvenient to obtain a specific frequency or wavelength that the MDM waveguide can only be statically modulated. Graphene, as a two-dimensional planar honeycomb structure can support the propagation of the SPPs in the mid-infrared and THz range, becomes the most promising candidate in many plasmonic materials owing to many excellent optical properties such as strong locality, low loss, near field enhancement, dynamic adjustability, etc [13, 14]. Consequently, graphene-based plasmonic optics has been used in many applications, for example, light-sensing [15, 16], absorption [17–19], switching [20], and other fascinating phenomena such as nonlinear optics [21, 22] and plasmon-induced transparency (PIT) [23–26]. The PIT effect, which is the result of destructive interference between the superradiation mode and the subradiation mode, has produced a variety of plasmonic applications, for example, plasmonic switching [20, 27], slow-light propagation [28], holographic imaging [29], and optical storage [30]. To achieve such a complex interaction between the light and the matter, the PIT can be obtained in heterogeneous graphene ribbons [31], single-layer or multi-layer graphene [32–34], and graphene-based metasurfaces [35]. However, these plasmonic devices are not only rather complicated in design, but also single-mode in terms of the modulation. Moreover, it is mainly that the resonance frequency will be tuned by manipulating the Fermi level of graphene in the modulation of most plasmonic devices. Since the transmittance of the PIT is neglected, the on-to-off modulation cannot be realized.

In this study, the proposed PIT metasurface, which consists of the periodic graphene ribbons and graphene strips, is easier to implement and fabricate. Through chemical vapor deposition (CVD) [36], the graphene ribbons and the graphene strips can be grown on the copper foil, which are transferred to a flat substrate by dry and wet transfer techniques. This technique produces fewer tears, cracks, and lower sheet resistance. Secondly, one of the most significant advantages is that the left (right) transmission dip is mainly affected by the gate voltage applied to graphene ribbons (stripes), respectively, meaning the dual-mode on-to-off modulation can be realized. Thirdly, even if the Fermi level of graphene is low, the absorption of the proposed metasurface can reach 50%, demonstrating an extraordinary absorber. Finally, when the mobility of the graphene ribbon and the graphene strip are both 3 m^2^/(Vs), the group delay can be as high as 0.7 ps, representing the proposed metasurface also has distinguished slow-light functions. Moreover, coupling effects between the graphene ribbons and the graphene strips in PIT metasurface with different structural parameters also are studied in detail. Therefore, this research lays a solid foundation for the dual-mode on-to-off multi-function modulator.

## Methods

**Fig. 1:**
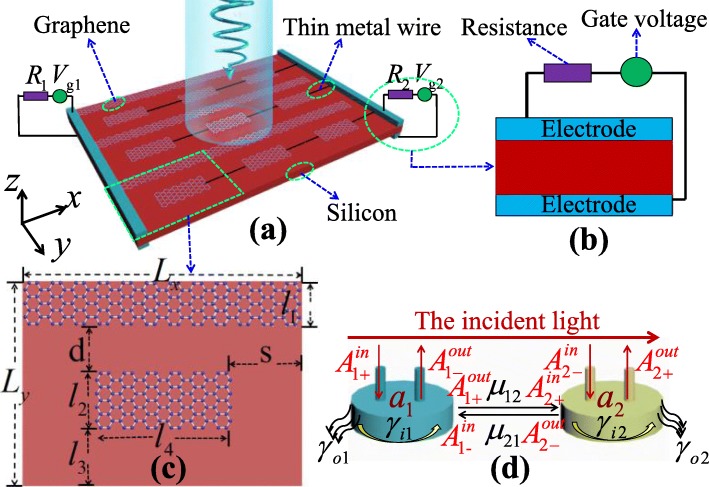
**a** Schematic of the 3 × 3 unit structure of the PIT metasurface. **b** The modulated diagram of gate voltage. **c** Top view of structural unit with geometric parameters *L*_*x*_ = 6.0 μm, *L*_*y*_ = 4.0 μm, *l*_1_ = 1.0 μm, *l*_2_ = 1.4 μm, *l*_3_ = *d* = 0.8 μm, *l*_4_ = 2.9 μm, and *S* = 1.55 μm. **d** Coupling diagram between the graphene ribbon and the graphene strip

The configuration of the PIT metasurface composed of the patterned single-layer graphene, the electrodes, the thin metal wires, and the substrate silicon is illustrated in Fig. 1a. The graphene ribbons are connected with the left electrode to modulate their Fermi levels by the gate voltage *V*_*g*1_. Moreover, the graphene strips are connected with the right electrode using thin metal wires, and a gate voltage *V*_*g*2_ is applied to modulate their Fermi levels [37, 38]. The gate voltages *V*_*g*1_ and *V*_*g*2_ can respectively modulate the Fermi levels of the graphene ribbons and the graphene strips to further realize the dual-mode modulation of the PIT. It is worth noting that the influence on the transmission effect can be ignored owing to the small size of the connecting wires [39]. In Fig. 1b, the Fermi level *E*_*f*_ of single-layer graphene can be indirectly modulated by the gate voltage, which can be expressed as [40]:

1 $$ {E}_f=\hslash {\upsilon}_F\sqrt{\frac{\pi {\varepsilon}_0{\varepsilon}_d{V}_{\mathrm{g}}}{e{d}_0}}. $$

Here, *ħ*, *ε*_*d*_, *ε*_0_, *e*, *d*_0_, and *v*_*F*_ are the reduced Planck constant, the static permittivity of silicon, the vacuum permittivity, the electron charge, the silicon thickness, and the Fermi velocity, respectively. It is worth mentioning that the carrier concentration as high as 4 × 10^18^ m^−2^ in graphene sheet was observed by employing an electrolytic gate, meaning *E*_*f*_ = 1.17 eV [41]; using this method, the Fermi energy level of graphene could be experimentally modified from 0.2 eV to 1.2 eV after applying a high bias voltage [42]. The structural unit of the proposed PIT metasurface, which consists of a graphene ribbon and a graphene strip placed on the substrate silicon, as illustrated in Fig. 1c. The periodicity is taken as *L*_*x*_ and *L*_*y*_; the coupling distance between the graphene ribbon and the graphene strip is *d*; the lateral displacement of the graphene strip is *S*.

The optical conductivity of a single-layer graphene sheet is mainly composed of inter-band and intra-band contributions [43–45], which can be expressed as

2 $$ \varepsilon \left(\omega \right)=1+\frac{\sigma_g}{\varepsilon_0\omega \varDelta}i. $$

3 $$ {\sigma}_g={\sigma}^{\mathrm{intra}}+{\sigma}^{\mathrm{inter}}. $$

4 $$ {\sigma}^{\mathrm{intra}}=\frac{2i{e}^2{k}_BT}{\pi {\hslash}^2\left(\omega +i{\tau}^{-1}\right)} In\left[2\cosh \left(\frac{E_f}{2{k}_BT}\right)\right]. $$

5 $$ {\sigma}^{\mathrm{inter}}=\frac{i{e}^2\left(\omega +i{\tau}^{-1}\right)}{4\pi {k}_BT}{\int}_0^{+\infty}\frac{G\left(\xi \right)}{\hslash^2{\left(\omega +i{\tau}^{-1}\right)}^2/{\left(2{k}_BT\right)}^2-{\xi}^2} d\xi . $$

Here, G(*ξ*) = sinh(*ξ*)/[cosh(*E*_*f*_ /*k*_*B*_*T*)+cosh*ξ*], where *ξ* = *ε*/*k*_*B*_*T*. Moreover, *ω*, *k*_*B*_, *σ*_*g*_, *σ*_inter_, and *σ*_intra_ are the angular frequency of incident light, the Boltzmann constant, the conductivity of single-layer graphene, the inter-band, and intra-band contributions, respectively. In this work, the room temperature is *T* = 300 K; the thickness of the graphene is Δ = 0.34 nm. *σ*_inter_ can be ignored owing to *k*_*B*_*T* ≪ 2*E*_*f*_ in the terahertz band. Thus, *σ*_*g*_ can be expressed as

6 $$ {\sigma}_g=\frac{i{e}^2{E}_f}{\pi {\hslash}^2\left(\omega +i{\tau}^{-1}\right)}. $$

Here, the electron relaxation time can be expressed as *τ* = *μ*_0_*E*_*f*_/(*ev*_*F*_^2^) [40], with *μ*_0_ = 1 m^2^/(Vs) being the graphene mobility. Besides, the propagation constant *β* of the incident light on the graphene surface can be expressed as [46]

7 $$ \frac{\varepsilon_1}{\sqrt{\beta^2-{\varepsilon}_1{k}_0^2}}+\frac{\varepsilon_2}{\sqrt{\beta^2-{\varepsilon}_2{k}_0^2}}=-\frac{i{\sigma}_g}{\omega {\varepsilon}_0}. $$

Here, *ε*_1_*, ε*_2_, and *k*_0_ are relative permittivity of silica and air, and the wave vector of the plane wave, respectively.

In Fig. 1d, the coupled-mode theory (CMT) [47] is used to fit the transmission and absorption spectra of FDTD numerical simulations. Elements A_1_ and A_2_ serve as two antennas to describe the coupling effect between the graphene ribbon and the graphene strip. When the incident light is illuminated from A and exited from B, the relation can be obtained by

8 $$ \left(\begin{array}{cc}{\gamma}_1& -i{\mu}_{12}\\ {}-i{\mu}_{21}& {\gamma}_2\end{array}\right)\cdot \left(\begin{array}{c}{a}_1\\ {}{a}_2\end{array}\right)=\left(\begin{array}{cc}-{\gamma}_{o1}^{1/2}& 0\\ {}0& -{\gamma}_{o2}^{1/2}\end{array}\right)\cdot \left(\begin{array}{c}{A}_{1+}^{in}+{A}_{1-}^{in}\\ {}{A}_{2+}^{in}+{A}_{2-}^{in}\end{array}\right). $$

Here, *γ*_1(2)_ *=* (*iω* – *iω*_1(2)_ – *γ*_*i*1(2)_ *– γ*_*o*1(2)_), in which the inter-loss coefficient is *γ*_*i*1(2)_ *= ω*_1(2)_/(2*Q*_*i*1(2)_) and the extra-loss coefficient is *γ*_*o*1(2)_ *= ω*_1(2)_ /(2*Q*_*o*1(2)_). Additionally, *Q*_*i*1(2)_ = Re(*n*_eff_)/Im(*n*_eff_) [29] is the inter-loss quality factor, which can be obtained by effective refractive index *n*_eff_ *= β*/*k*_*0*_. The intra-loss quality factor can be obtained by 1/*Q*_*t*1(2*)*_ = 1/*Q*_*i*1(2)_ + 1/*Q*_*o*1(2)_, with Q_*t*1(2)_ = *f*/Δ*f* being the quality factor of the whole system (Δ*f* is 3 dB bandwidth). Following the conservation of energy, coupling relationship between two antennas is as follows:

9 $$ {A}_{2+}^{\mathrm{in}}={A}_{1+}^{\mathrm{out}}{e}^{i\varphi},{A}_{1-}^{\mathrm{in}}={A}_{2-}^{\mathrm{out}}{e}^{i\varphi}, $$

10 $$ {A}_{1+}^{\mathrm{o}\mathrm{ut}}={A}_{1+}^{\mathrm{in}}-a{\gamma}_{\mathrm{o}1}^{1/2},{A}_{2+}^{\mathrm{o}\mathrm{ut}}={A}_{2+}^{\mathrm{in}}-b{\gamma}_{o2}^{1/2}, $$

11 $$ {A}_{1-}^{\mathrm{o}\mathrm{ut}}={A}_{1-}^{\mathrm{in}}-a{\gamma}_{\mathrm{o}1}^{1/2},{A}_{2-}^{\mathrm{o}\mathrm{ut}}={A}_{2-}^{\mathrm{in}}-b{\gamma}_{o2}^{1/2}, $$

12 $$ {A}_{2-}^{\mathrm{in}}=0. $$

Here, the subscripts “+” and “–” represent that the antennas are illuminated in the same and opposite directions; the superscripts “in” and “out” represent the sign of the incident light entering and exiting the antennas. In addition, *μ*_nm_ (*n* = 1, 2, *m* = 1, 2, *n* ≠ *m*) and *φ* are the coupling coefficients and the phase difference between two antennas, respectively. Thus, we can obtain the transmission coefficient and the reflection coefficient of the proposed PIT metasurface.

13 $$ t=\frac{A_{2+}^{out}}{A_{1+}^{i n}}={e}^{i\varphi}+\left[{\gamma}_{o1}{\gamma}_2{e}^{i\varphi}+{\gamma}_{o2}{\gamma}_1+{\left({\gamma}_{o1}{\gamma}_{o2}\right)}^{1/2}\left({\chi}_1{e}^{i\varphi}+{\chi}_2\right)\right]\cdot {\left({\gamma}_1{\gamma}_2-{\chi}_1{\chi}_2\right)}^{-1}, $$

14 $$ r=\frac{A_{1-}^{out}}{A_{1+}^{i n}}=\left[{\gamma}_{o1}{\gamma}_1+{\gamma}_{o2}{\gamma}_1{e}^{i\varphi}+{\left({\gamma}_{o1}{\gamma}_{o2}\right)}^{1/2}\left({\chi}_1+{\chi}_2{e}^{i\varphi}\right)\right]\cdot {\left({\gamma}_1{\gamma}_2-{\chi}_1{\chi}_2\right)}^{-1}. $$

Where *χ*_1(2)_ = *iμ*_12(21)_ +(*γ*_*o*1(2)_*γ*_*o*2(1)_)^1/2^*e*^*iφ*^. Then, the transmission and absorption of the proposed PIT metasurface can be obtained by

15 $$ T={t}^2,A=1-{t}^2-{r}^2. $$

## Results and Discussion

Very recently, the graphene ribbons, as one of the most promising candidates in the graphene series owing to the fact that they are greatly easy to achieve experimentally and can support localized plasmons (mainly based on Fabry-Perot-like standing wave resonance) [48–50] and propagate plasmons [51, 52], have attracted a lot of attention in the field of nanophotonics. Here, we exploit the plasmonic coupling between the graphene ribbons and the graphene strips to demonstrate an excellent PIT effect.

**Fig. 2:**
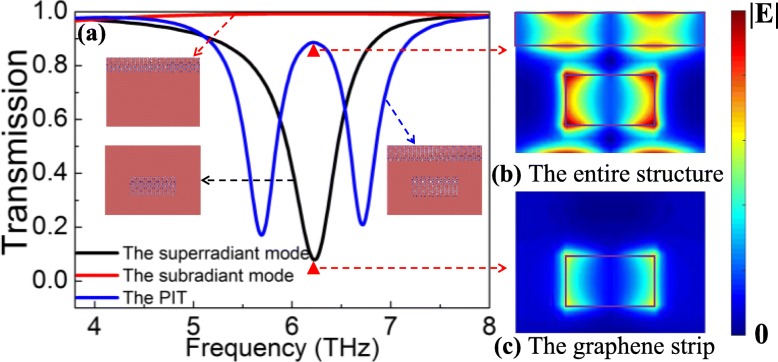
**a** Simulated transmission spectra of three graphene metasurfaces. **b** Electric field distribution of the entire structure at the resonance peak. **c** Electric field distribution of the graphene strip at the resonance dip. Here, *E*_*f*1_ = *E*_*f*2_ = 1.0 eV

So as to discuss the physical origin of the PIT effect, simulated transmission spectra of three graphene metasurfaces and electric field distributions of the entire structure and graphene strip at the resonance frequency are illustrated in Fig. 2a–c. In Fig. 2a, when the metasurfaces are shone by the x-polarized light, a subradiant mode can be excited in the graphene ribbon, which produces a red curve with a transmittance of 1. Meanwhile, a superradiant mode can be directly excited in the graphene strip, which brings about a black Lorentz curve with a transmission dip of 7.90%. As a result, the subradiant mode can be indirectly excited by the superradiant mode, forming a blue PIT curve with a transmission peak of 88.61% generated by the entire structure. In addition, electric field distributions of the entire structure and the graphene strip at the resonance frequency also can explain the physical origin of the PIT phenomenon. When only the graphene strips exist in the structural units of each pattern graphene metasurface, the electric field energy around the graphene strip is in an equilibrium state, as illustrated in Fig. 2c. In this case, only the weaker electric field is confined around the graphene strip, which produces a Lorentz curve with a lower quality factor. However, when a graphene ribbon is added to the metasurface, the electric field balance around the graphene strip is broken. At the moment, since the coupling effect between them, the electric field around the graphene strip is enhanced, and the graphene ribbon is also excited by the near field, as illustrated in Fig. 2b. Therefore, the electric field energy is localized around the graphene strip and the graphene ribbon surface, forming a PIT curve with higher quality factors.

**Fig. 3:**
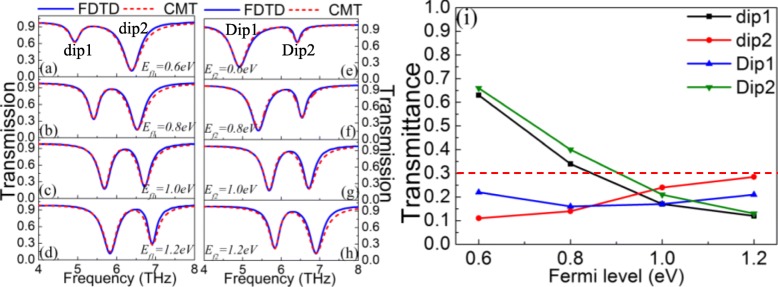
Transmission spectra of FDTD simulation and CMT fitting (**a**–**d**) for different *E*_*f*1_ when *E*_*f*2_ = 1.0 eV. **e**–**h** For different *E*_*f*2_ when *E*_*f*1_ = 1.0 eV. **i** Relationship between the transmittance of resonance dip and the Fermi level

**Fig. 4:**
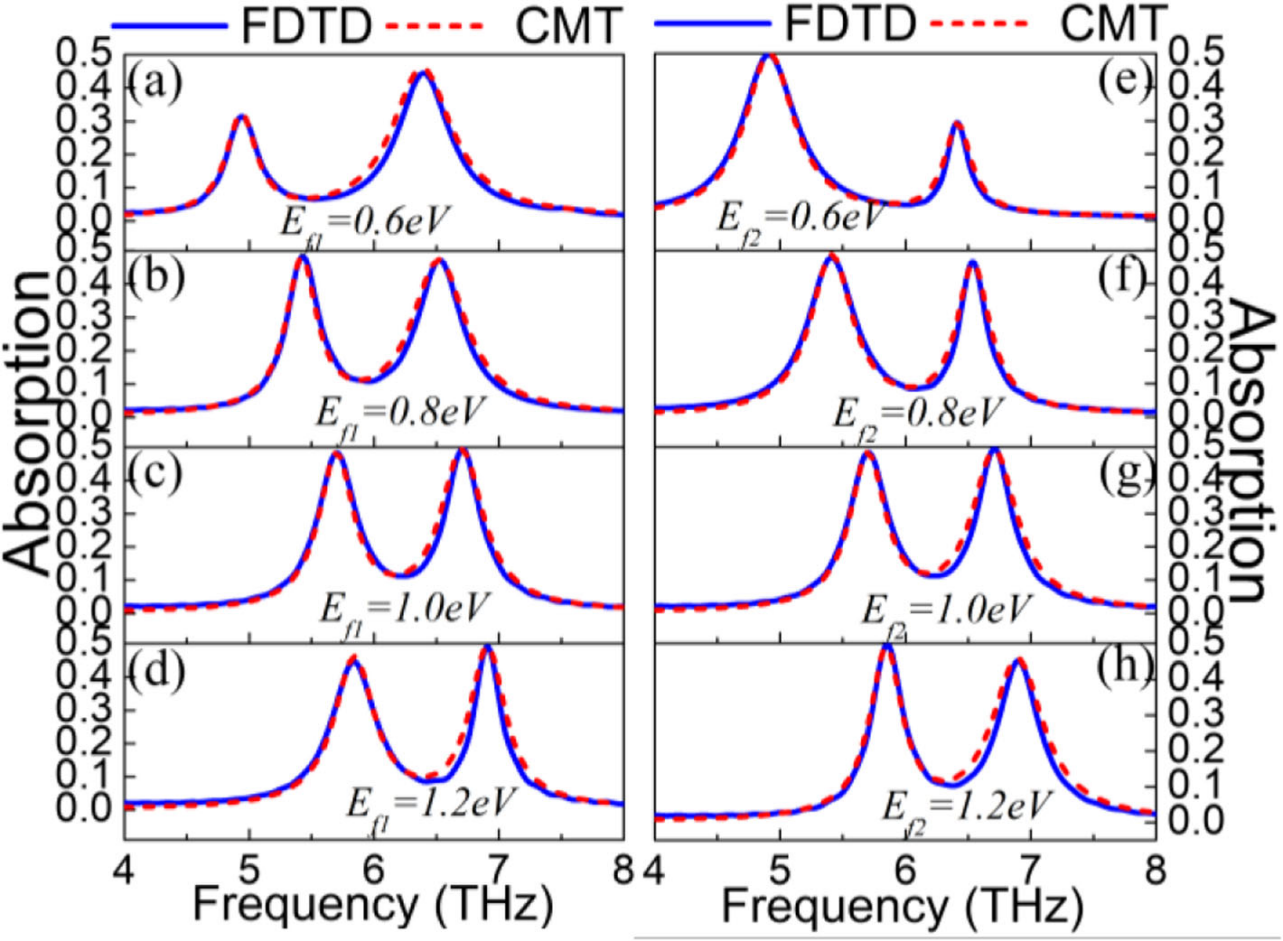
Absorption spectra of FDTD simulation and CMT fitting (**a**–**d**) for different *E*_*f*1_ when *E*_*f*2_ = 1.0 eV. **e**–**h** For different *E*_*f*2_ when *E*_*f*1_ = 1.0 eV

The dual-mode on-to-off modulation of PIT can be achieved by two gate voltages applied to the graphene ribbons and the graphene strips, as illustrated in Figs. 3a–h. Here, the four resonance dips are labeled “dip1, dip2, Dip1, Dip2.” When the Fermi level *E*_*f*2_ of the graphene strip is fixed at 1.0 eV, the Fermi level *E*_*f*1_ of the graphene ribbon is changed to explore the PIT effect. In Fig. 3a–d, as the Fermi level *E*_*f*1_ increases from 0.6 eV to 1.2 eV, there is a significant change in dip1. For one thing, the transmittance of the dip1 is remarkably reduced indicating that an on-to-off modulation can be obtained. For another, the dip1 has an obvious blue-shift demonstrating that it is sensitive to the change of Fermi level *E*_*f*1_ and can realize frequency modulation. Besides, when the Fermi level *E*_*f*1_ of the graphene ribbon is fixed at 1.0 eV, a similar phenomenon occurs in Dip2 with the increase of the Fermi level *E*_*f*2_. However, the blue-shift is more significantly observed in the left dip in both cases. When Fermi levels of the graphene strip and the graphene ribbon are both 1.0 eV, the resonance frequency of the superradiation mode and the monopole resonance frequency of the subradiation mode are basically 6.2 THz. Thus, the coupling between them forms a symmetrical PIT. When the Fermi level *E*_*f*1_ of the graphene ribbon is increased from 0.6 eV to 1.0 eV, the monopole resonance frequency of the subradiation mode shifted from the left side to the 6.2 THz due to the change of the graphene ribbon conductivity. In the case, the coupling between the subradiation mode and the superradiation mode is weak owing to different resonance frequencies, generating a highly asymmetric PIT. The obvious blue-shift of the dip1 in Fig. 3a–d is mainly affected by the blue-shift of the sub-radiation mode. Similarly, the obvious blue-shift of the Dip1 in Fig. 3e–h is mainly influenced by the blue-shift of the superradiation mode. The detailed on-to-off mechanism is illustrated in Fig. 3i. In the design of the on-to-off modulator, the “on” is set to a transmittance exceeding 0.3; otherwise, it is set to the “off.” Thus, the proposed PIT metasurface can realize the dual-mode-on function in the Fermi level of 0.6 eV to 0.8 eV and the dual-mode-off function in the Fermi level of 0.8 eV to 1.2 eV. In short, the gate voltage *V*_*g*1_ mainly regulates the left transmission dip, yet the right transmission dip is chiefly tailored by the gate voltage *V*_*g*2_. Therefore, a dual-mode on-to-off modulator is realized. Meanwhile, the dual-mode modulation of plasmon-induced absorption (PIA) is also obtained in Fig. 4a–h. With the increase of the Fermi level, the PIA has a clear blue-shift. Even if the Fermi level of graphene is low, the absorption of the proposed metasurface can reach 50%. This is because graphene is similar to loss properties when the Fermi level is low, resulting in high loss and absorption [53]. The phenomenon means that lower Fermi level can achieve a higher absorption, thereby reducing the required voltage. Furthermore, the transmission and absorption spectra of FDTD simulation both are fitted by CMT. Here, the blue curve indicates the FDTD simulation result; the red dotted curve indicates the CMT fitting data.

**Fig. 5:**
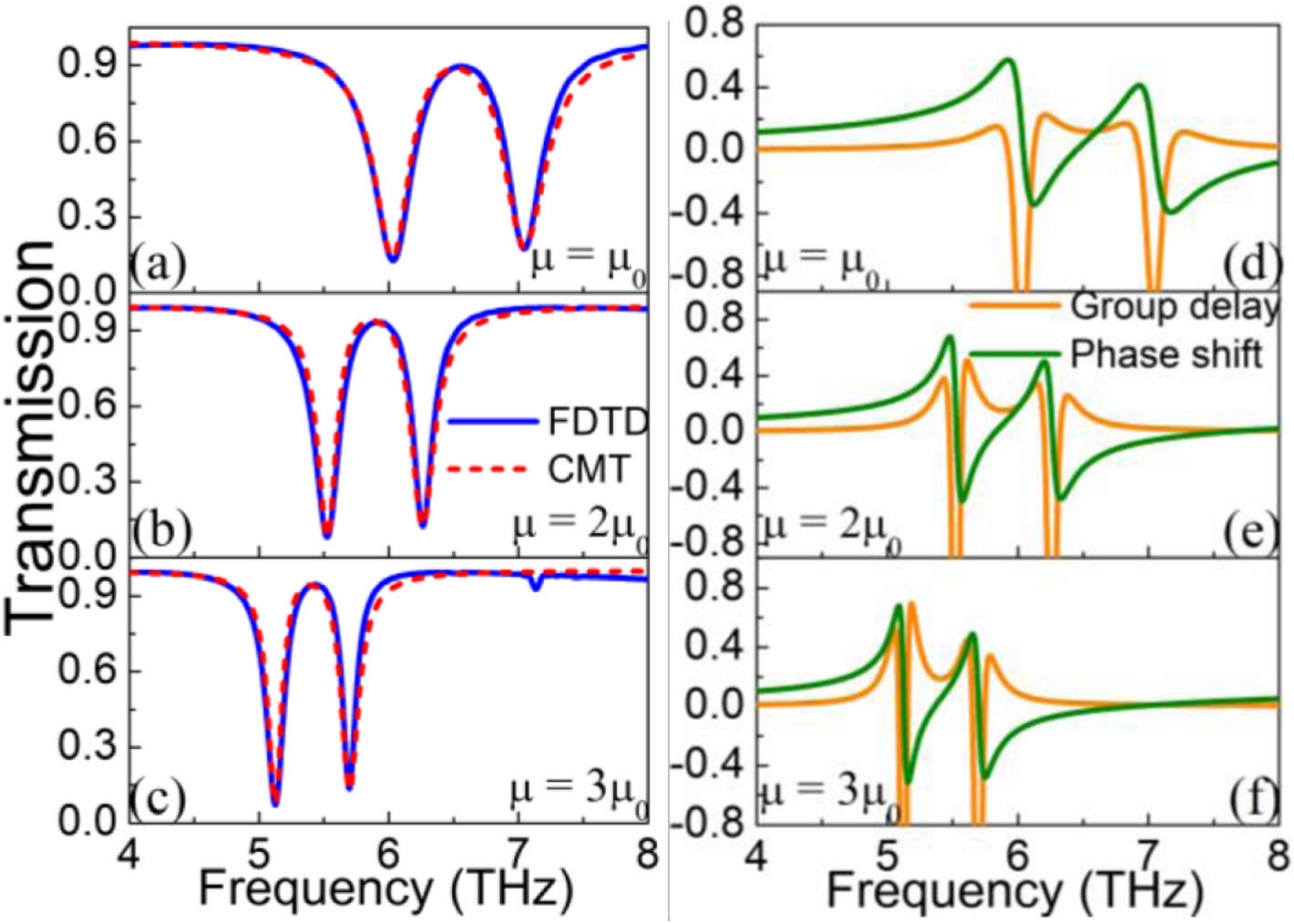
**a–c** Transmission spectra of FDTD simulation and CMT fitting with different graphene mobility *μ = μ*_0_, 2*μ*_0_, 3*μ*_0_. **d, e** Transmission phase shift and the group delay with different graphene mobility *μ = μ*_0_, 2*μ*_0_, 3*μ*_0_. Here, *E*_*f*1_ = *E*_*f*2_ = 1.2 eV

In addition, transmission spectra with different graphene mobilities are also studied, as illustrated in Fig. 5(a-c). A fully symmetrical PIT curve is obtained when *E*_*f*1_ = *E*_*f*2_ = 1.0 eV. On this basis, the graphene mobility is increased from 1.0 m^2^/(Vs) to 3.0 m^2^/(Vs) in a 1.0 m^2^/(Vs) step. As the graphene mobility increases, not only the transmission spectra show apparent red-shift, but also the 3 dB bandwidth of the transmission dips becomes narrower, meaning graphene mobility can also be used to dynamically modulate the PIT and quality factors of transmission dips. Here, transmission spectra of FDTD simulation and CMT fitting are still perfectly matched. It is known that the performances of the slow-light effect are better with the higher quality factor of the transmission dip. Therefore, the transmission phase shift and the group delay with different graphene mobilities are plotted in Fig. 5d–e. The group delay is achieved by [54]:

16 $$ {\mathrm{t}}_g=\frac{d\phi \left(\omega \right)}{d\omega}, $$

where *ϕ*(*ω*) is the phase shift calculated by *ϕ*(*ω*) =  *arg* (*t*). The results show that both the group delay and the phase shift are 0 when the transmittance of the system is close to 1. Moreover, the large group delay occurs at the transmission peak and its surroundings owing to the fact that the graphene ribbon and the graphene strip have a strong coupling effect at the resonance frequency. When the graphene mobility reaches 3*μ*_0_, the group delay of the system can be as high as 0.7 ps. However, the group delay at the transmission dips reaches a large negative value, meaning fast light propagation in the system. Meanwhile, the phase shift has also changed dramatically at the transmission dips. Zhang et al. recently has proposed an absorption efficiency of 50% and slow-light performance with a patterned graphene structure [25]. However, the proposed structure unit composed of graphene double strips and a graphene ribbon, which is more complex, cannot realize the dual-mode on-to-off and absorption modulation. Besides, it is unreasonable to analyze the absorption efficiency by changing the mobility of the graphene double strips with only the graphene ribbon being applied with a gate voltage. Furthermore, slow-light effect analyzed by the group index which is largely dependent on the thickness of the substrate is not objective. And the group index which can only reach 382 is poor.

**Fig. 6:**
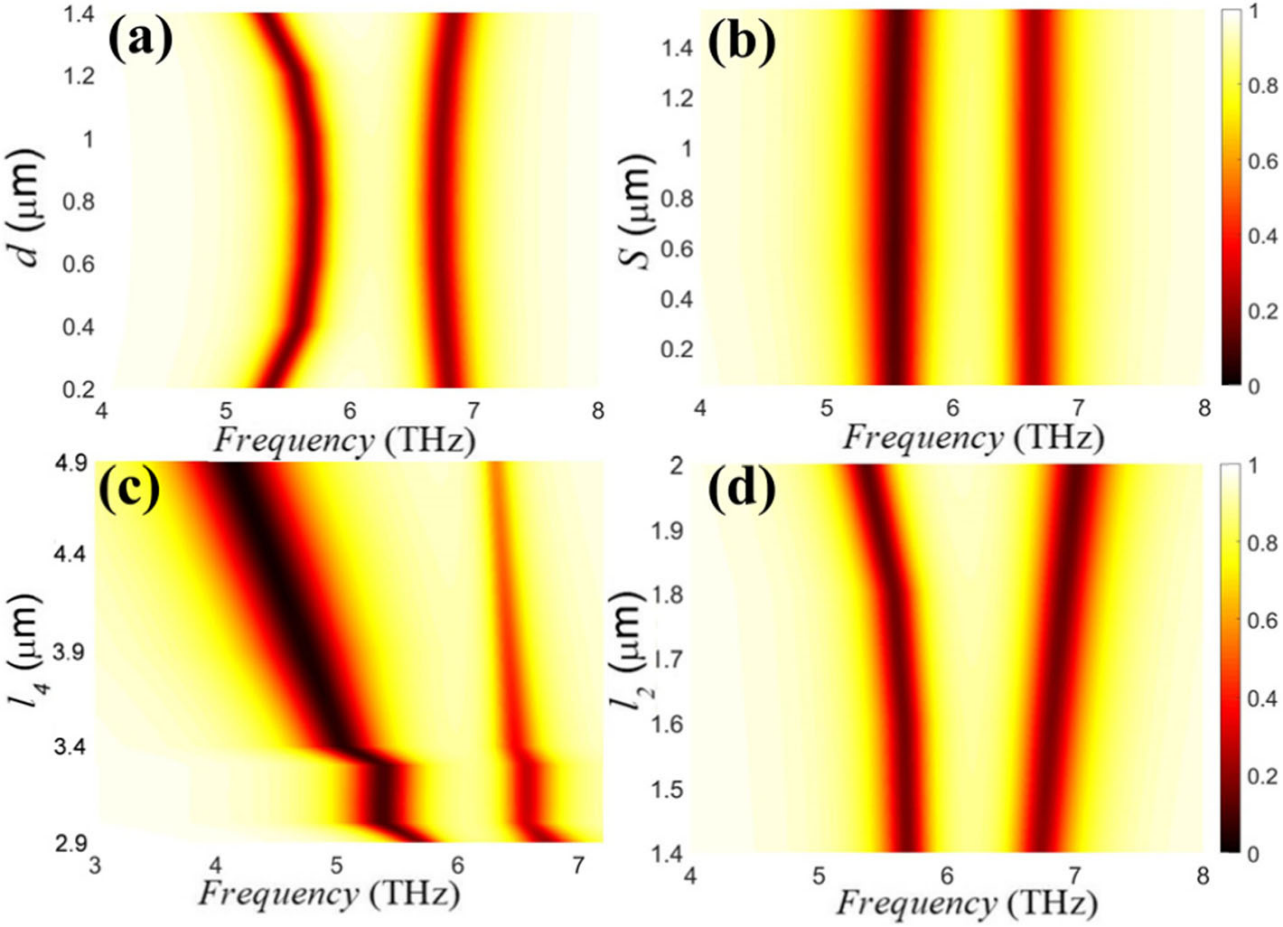
Transmission spectra dependence on different geometric parameters. **a** The coupling distance, *d*, (**b**) the lateral displacement, *S*, (**c**) the length of graphene strip, *l*_4_, (**d**) the width of graphene strip, *l*_2_

Finally, coupling effects between the graphene ribbons and the graphene strips in PIT metasurface with different structural parameters are studied in detail, as illustrated in Fig. 6a–d. Other structural parameters are based on Fig. 2a. From Fig. 6a, as the coupling distance increases, the left transmission dip is first blue-shifted and then red-shifted, while the right transmission dip is basically unchanged, meaning a change of the coupling distance has a greater influence for the left transmission dip. When the lateral displacement of the graphene strip increases, the position of the transmission dips does not change owing to the x-polarized incident light, as observed in Fig. 6b. Interestingly, in Fig. 6c, the increase of *l*_4_ results in stepped red-shift in the left transmission dip and its quality factor is getting smaller, indicating the dependence of the graphene strip length on left transmission spectra. Fig. 6d illustrates the increase of the graphene strip width causes a slight red-shift in the left transmission dip and a slight blue-shift in the right transmission dip, increasing distance between the transmission dips. It is worth mentioning that since the increments of the length and width for the graphene strip improve the inductance of the resonant system, a significant phenomenon is generated.

## Conclusion

In short, we have numerically simulated and theoretically calculated the PIT in the patterned metasurface composed of the graphene ribbons and the graphene strips, which is caused by destructive interference between the superradiant mode and the subradiant mode. Interestingly, the dual-mode on-to-off modulation of PIT can be achieved by two gate voltages applied to the graphene ribbons and the graphene strips. Moreover, an absorption rate of 50% and slow-light property of 0.7 ps are achieved, demonstrating the proposed PIT metasurface has important applications in absorption and slow-light. Furthermore, coupling effects between the graphene ribbons and the graphene strips in PIT metasurface with different structural parameters are studied in detail. Thus, this work provides potential applications for the implementation of dual-mode on-to-off multi-function modulators.

## Data Availability

All data generated or analyzed during this study are included in this published article.

## Abbreviations

CMT: Coupled mode theory
CVD: Chemical vapor deposition.
FDTD: Finite-difference time-domain
MDM: Metal-dielectric-metal
PIT: Plasmon-induced transparency
SPPs: Surface plasmon polaritons
